# Portal venous gas accompanied by gallbladder torsion: a case report

**DOI:** 10.1093/jscr/rjac491

**Published:** 2022-10-31

**Authors:** Takayuki Tohma, Yoshiro Kobe, Mitsuhiko Yoshida, Masaya Ushio

**Affiliations:** Department of Acute Care Surgery, Chiba Emergency Medical Center, Chiba, Japan; Department of Acute Care Surgery, Chiba Emergency Medical Center, Chiba, Japan; Department of Acute Care Surgery, Chiba Emergency Medical Center, Chiba, Japan; Department of Acute Care Surgery, Chiba Emergency Medical Center, Chiba, Japan

## Abstract

Portal venous gas (PVG) generally suggests critically ill conditions such as severe bowel ischemia. We herein report a rare case of gallbladder torsion with PVG. An 88-year-old woman complained of right hypogastric pain. Ultrasonography (US) showed diffuse wall thickening of her gallbladder and mobile echogenic foci moving inside the portal venous branches. Computed tomography showed a thickened wall of the gallbladder with poor enhancement and tiny pockets of air in the portal venous branches (segments 4 and 5). There was no evidence of other visceral ischemia. She was diagnosed with necrotic cholecystitis and immediately underwent an emergency operation. We found a gangrenous gallbladder with 180° clockwise rotation along the longitudinal axis and performed cholecystectomy. We confirmed the disappearance of PVG with US after the operation. Her postoperative course was uneventful. Gallbladder diseases can produce PVG, and US might be a useful diagnostic modality to evaluate changes in PVG.

## INTRODUCTION

Portal venous gas (PVG) is a diagnostic finding with a poor prognosis [1]. It has been considered to be a critical condition, with most cases inducing massive bowel necrosis due to superior mesenteric arterial embolism and non-occlusive mesenteric ischemia [2]. We herein report a rare case of PVG associated with a gallbladder torsion.

### CASE REPORT

An 88-year-old female patient who had been hospitalized in a rehabilitation hospital due to lumbar compression fracture suddenly complained of right hypogastric pain. She was transferred to another hospital and diagnosed with acute cholecystitis. Finally, she was referred to our institution for further treatment.

**Figure 1:**
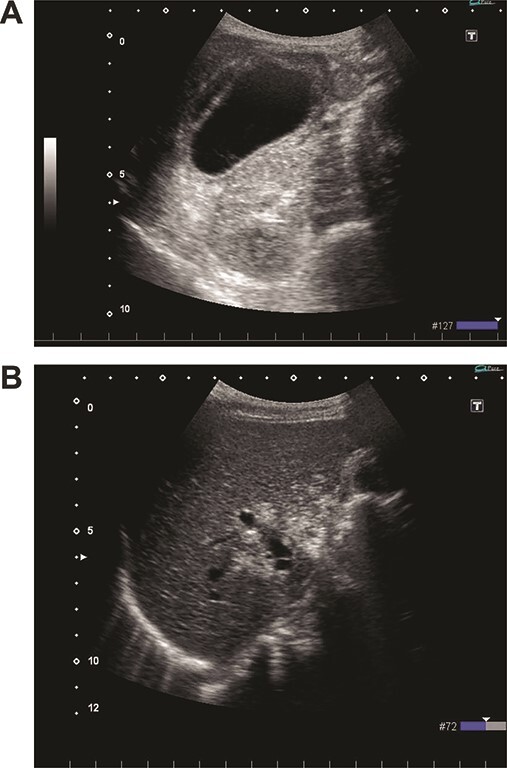
(**A**) Ultrasonography shows distention and wall thickening of the gallbladder. (**B**) Ultrasonography shows small mobile echogenic foci and hyperechogenic linear structures in the middle segment and right anterior segment of the liver.

**Figure 2:**
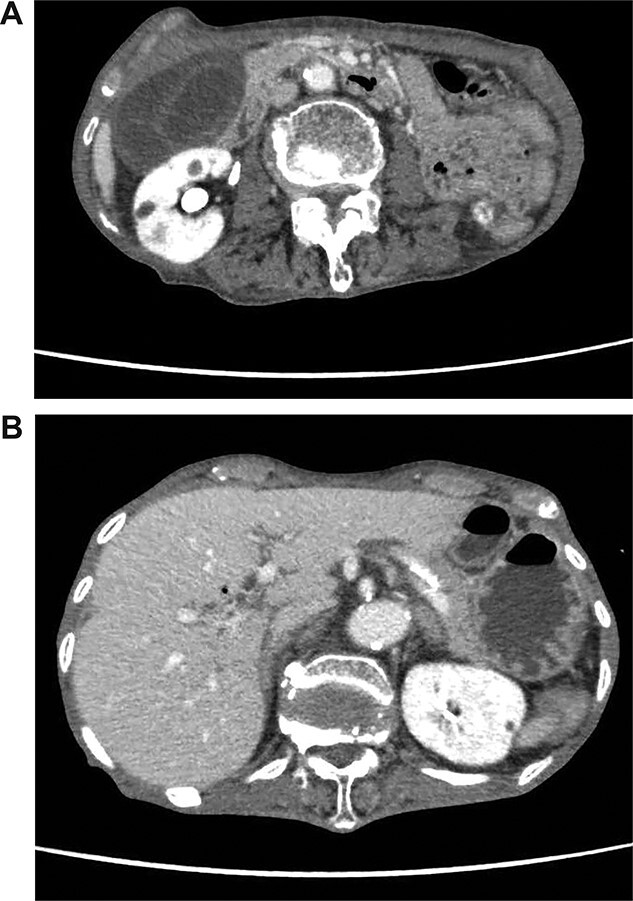
(**A**) Abdominal CT shows a poorly enhanced and thickened wall of the gallbladder. (**B**) Abdominal CT shows tiny bubbles in the intrahepatic portal venous branches.

On admission, her vital signs were normal. Her medical history included dermatomyositis, hypertension and osteoporosis. She had a white blood cell count of 10.6 × 10^3^/μL, hemoglobin of 8.2 g/dL and lactic acid of 0.8 mmoL/L. A physical examination demonstrated strong tenderness in her right upper quadrant abdomen. Ultrasonogramphy (US) showed diffuse wall thickening of her gallbladder without cholecystolithiasis. US also revealed small mobile echogenic foci moving inside the portal venous branches (Fig. 1). The hyperechogenic foci were seen in the middle segment and right anterior segment of the liver. Contrast-enhanced computed tomography (CT) showed a diffusely thickened wall of the gallbladder with poor enhancement, suggesting acute necrotizing cholecystitis (Fig. 2). Tiny bubbles were seen in the intrahepatic portal venous branches (segment 4 and segment 5), but there was no evidence of bowel ischemia. She was then diagnosed with necrotic cholecystitis and immediately underwent emergency operation.

**Figure 3:**
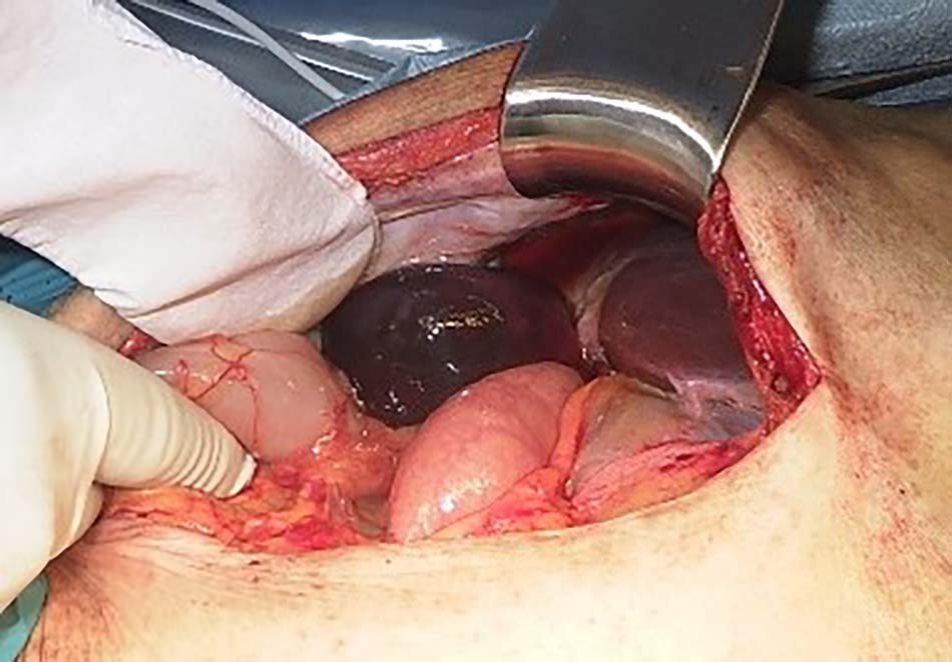
Intraoperative finding shows a gangrenous gallbladder with 180° clockwise rotation.

**Figure 4:**
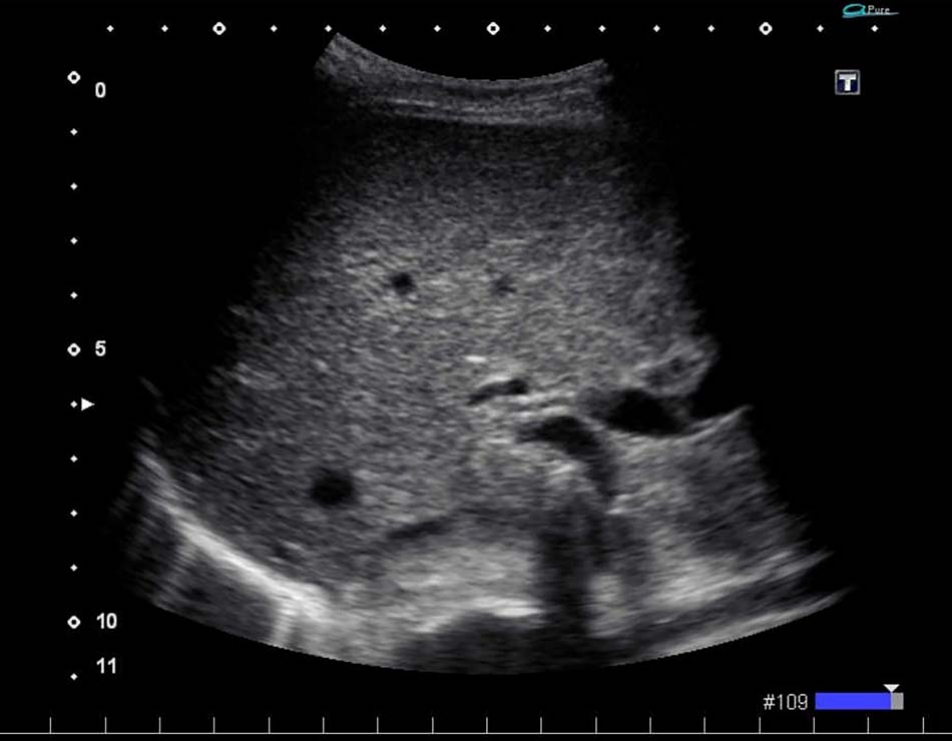
Ultrasonography after surgery shows no findings of PVG in the liver.

We found a gangrenous gallbladder with 180° clockwise rotation along the longitudinal axis and performed cholecystectomy (Fig. 3). Intraoperative exploration revealed no ischemic findings in other visceral organs. On US obtained immediately after the operation, the hyperechogenic foci disappeared (Fig. 4). Her postoperative course was uneventful, and she was transferred to the rehabilitation hospital on the 12th day. Histology confirmed a necrotic gallbladder without neoplastic changes.

## DISCUSSION

PVG is recognized as a critical sign with a high mortality rate [2]. Wolfe and Evans first reported six infant cases under fetal conditions [3]. Recent advances in diagnostic modalities, such as CT and US, have made it possible to detect even a small amount of PVG [4]. As a result, the number of cases with non-critical PVG has increased [5]. However, most patients with PVG still have a high mortality rate and require immediate surgical intervention [6].

The emergent treatments for PVG depend on the etiology [7]. Surgeons have to remove the origin of PVG to save patients, as they must detect the source of PVG. Most cases of surgical PVGs are supplied from mesenteric veins of ischemic bowels. We experienced a rare case of gallbladder torsion. Anatomically, cystic veins usually drain to intrahepatic portal veins [8]. Therefore, PVG can also occur in ischemic gallbladder diseases, such as necrotizing cholecystitis, emphysematic cholecystitis and gallbladder torsion [9]. Gallbladder diseases should thus be considered in the differential diagnosis of PVGs in addition to bowel ischemic diseases.

In the present case, US revealed tiny pockets of gas that disappeared immediately after surgery. Chevallier *et al*. reported that US might have higher detectability than CT [10]. In general, PVGs are said to be distributed in accordance with gravity and gather at the surface of the liver [11]. However, the gas in the current case was detected around the hepatic hilum. This may be because cystic veins usually drain to the portal venous system via intrahepatic portal branches of segments 4 and 5 [12]. In cases of gallbladder disease, PVG can appear around the hepatic hilum instead of at the liver surface, which might be a useful finding when differentiating the condition from bowel ischemia.
